# Effect of Adding a Virtual Community (Bulletin Board) to Smokefree.gov: Randomized Controlled Trial

**DOI:** 10.2196/jmir.1124

**Published:** 2008-12-19

**Authors:** Jacqueline L Stoddard, Erik M Augustson, Richard P Moser

**Affiliations:** ^3^National Cancer Institute/DCCPS/Behavioral Research ProgramRockvilleMDUSA; ^2^National Cancer Institute/DCCPS/BRP/Tobacco Control Research BranchRockvilleMDUSA; ^1^SAIC-Frederick IncNCI-FrederickFrederickMDUSA

**Keywords:** Smoking cessation, Internet, World Wide Web, randomized trial, self-help

## Abstract

**Background:**

Demand for online information and help exceeds most other forms of self-help. Web-assisted tobacco interventions (WATIs) offer a potentially low-cost way to reach millions of smokers who wish to quit smoking and to test various forms of online assistance for use/utilization and user satisfaction.

**Objectives:**

Our primary aim was to determine the utilization of and satisfaction with 2 versions of a smoking cessation website (smokefree.gov), one of which included an asynchronous bulletin board (BB condition). A secondary goal was to measure changes in smoking behavior 3 months after enrollment in the study.

**Methods:**

All participants were adult federal employees or contractors to the federal government who responded to an email and indicated a willingness to quit smoking in 30 days. We randomly assigned participants to either the BB condition or the publicly available version—usual care (UC)—and then assessed the number of minutes of website use and satisfaction with each condition as well as changes in smoking behavior.

**Results:**

Among the 1375 participants, 684 were randomized to the BB intervention, and 691 to the control UC condition. A total of 39.7% returned a follow-up questionnaire after 3 months, with similar rates across the two groups (UC: n=279, 40.3%; BB: n=267, 39.0%). Among those respondents assigned to the BB condition, only 81 participants (11.8%) elected to view the bulletin board or post a message, limiting our ability to analyze the impact of bulletin board use on cessation. Satisfaction with the website was high and did not differ significantly between conditions (UC: 90.2%, BB: 84.9%, *P*= .08). Utilization, or minutes spent on the website, was significantly longer for the BB than the UC condition (18.0 vs 11.1, *P* = .01) and was nearly double for those who remained in the study (21.2) than for those lost to follow-up (9.6, *P*< .001). Similar differences were observed between those who made a serious quit attempt versus those who did not (22.4 vs 10.4, *P*= .02) and between those with a quit date on or a few days prior to the enrollment date versus those with a later quit date (29.4 vs 12.5, *P* = .001). There were no statistically significant differences in quit rates between the BB and UC group, both in intent-to-treat analysis (ITT) and in analyzing the adherence subgroup (respondents) only. Combined across the UC and BB groups, 7-day abstinence was 6.8% with ITT and 17.6% using only participants in the follow-up (adherence). For participants who attempted to quit within a few days of study entry (vs 30 days), quit rates were 29.6% (ITT) and 44.4% (adherence).

**Conclusions:**

Quit rates for participants were similar to other WATIs, with the most favorable outcomes demonstrated by smokers ready to quit at the time of enrolling in the trial and smokers using pharmacotherapy. Utilization of the asynchronous bulletin board was lower than expected, and did not have an impact on outcomes (quit rates). Given the demand for credible online resources for smoking cessation, future studies should continue to evaluate use of and satisfaction with Web features and to clarify results in terms of time since last cigarette as well as use of pharmacotherapy.

**Trial Registration:**

Clinicaltrials.gov NCT00245076; http://clinicaltrials.gov/ct2/show/NCT00245076 (Archived by WebCite at http://www.webcitation.org/5dBuBASA0)

## Introduction

More than 4.8 million people are estimated to die each year from smoking-related disease worldwide [1]. In the United States, 44.2% of smokers try to quit each year [1], and about 10% of the adult population has searched online for information about quitting smoking [2]. Such demand dwarfs the 1% to 2% of smokers who call quitlines [3] and the 1.3% of smokers estimated to seek behavioral counseling each year [4,5]. The willingness of smokers to search for assistance online is widely attributedto the convenience and anonymity of the Internet. The reach of the Internet suggests that even if the direct effects of Web-assisted tobacco interventions (WATIs) are very small, a sizable population-level impact on smoking is achievable. However, studies of WATIs are challenged by low retention of participants and the massive sample sizes needed to capture small effects [6-9]. In addition, the difficulty of testing a “real-world” intervention [7] and the impracticality of constraining samples to those not using any other form of help contribute to the general reluctance in the field to employ a randomized controlled trial (RCT) to explore any direct effects of a Web-assisted intervention. However, the reach of the medium, its ease of use, and the many types of assistance that it can offer make it an excellent format in which to evaluate competing forms of help.

For example, the American Cancer Society (ACS) compared quit rates on their static website to 5 other interactive websites and found no differences among the 6451 people who participated [9]. However, when analyses were grouped by level of use, participants of interactive websites with higher utilization had slightly higher 7-day abstinence rates at 3 months (12.2% vs 10.2%) than participants of low-utilization websites. Similarly, in another RCT that included bupropion and frequent counseling before randomizing participants to an intensive website requiring log-ins or to no website [6], no direct effect of Web access was observed. However, as with the ACS trial, higher abstinence rates were reported by those who logged in most often. A third RCT that failed to show an effect of an intervention on quit rates compared a cessation website that emphasized mood management versus standard cessation materials [8]. While no difference between website conditions was observed, a unique benefit of mood management was shown for smokers with a past history of depression, consistent with previous research from this group [10]. One RCT that appeared to show an effect of one website over another was conducted by Strecher and colleagues [11]. This study reported higher 3-month quit rates among participants randomized to a website with tailored information versus untailored information. However, because participants in the tailored condition had more contact with study personnel than in the untailored condition, the effects could not be ascribed to the intervention. The only RCT that we know of to clearly demonstrate a difference between two similar websites was conducted by Etter [12], who randomized nearly 12,000 participants to receive either his original website (stoptabac.ch) or an abbreviated version of this website designed for Novartis that emphasized nicotine replacement information. Quit rates at 3 months (7-day abstinence) favored the original program both for current smokers (10.9% vs 8.9%) and former smokers (25.2% vs 15.7%).

Although results from these 5 studies are somewhat inconsistent, 2 commonalities were demonstrated. First, all showed that RCTs can be successfully conducted via the Internet and can produce abstinence rates comparable to many traditional cessation interventions. Second, these trials also highlight the importance of enrolling large numbers of participants (thousands) and ensuring that a large proportion of participants will actually use the Web feature being tested. In 2 of the above studies, insufficient utilization of the feature being tested may have prevented an effect from being observed [6,8]. Despite this, these RCTs provided useful information about different features of a website without the timing confound that occurs with serial testing.

RCTs that test the therapeutic effects of virtual communities on outcomes are scarce and have produced no evidence of direct effects on smoking outcomes [13]. In fact, we know of no studies showing that such tools favorably impact website usage or patterns of website use. However, previous descriptive studies outside of the tobacco control literature support the function of bulletin boards as providing social support through information exchange with others [14,15]. One study within the field of tobacco research found that participants of a widely used website for smoking cessation used the bulletin board more than any other feature on the website and that those who used it were 3 times more likely to be quit compared to those who did not [7].

In previous usability tests of smokefree.gov [16] and a customer satisfaction survey of this website (unpublished data, National Cancer Institute), the majority of registered participants (61%, n = 1261) agreed or strongly agreed that a bulletin board or similar feature would be valuable. Given this, we opted to test the usage and satisfaction with such a feature, including potential interaction effects with other features on the site, in the context of an RCT of smokefree.gov. We further sought to compare our intervention results (overall) to other similar studies. Finally, we examined demographic characteristics of our population to ensure that we had reached as broad a cross-section of smokers as was possible among smokers employed by the federal government.

## Methods

### Participants

Approximately 120,000 invitations to review the smokefree.gov website were sent out in 2 phases. All invitations were sent blind to the receiver’s smoking status; thus the majority was sent to nonsmokers. The first group of approximately 43,000 federal employees received an email invitation between April 12, 2005 and May 5, 2005 asking them to participate. The second series of emails was sent to a different group of approximately 80,000 federal employees and contractors between February 28, 2006 and November 11, 2006. Emails contained information about a service for smokers interested in quitting, along with an embedded link redirecting interested participants to a site used to screen for eligibility. The redirected page screened for eligibility and admitted those who indicated that they were a federal employee or contractor, were a minimum of 18 years old, and had a willingness to quit smoking. Federal employees and contractors were selected because federal agencies may not survey the public except under extraordinary circumstances (Paperwork Reduction Act of 1980, 44 USC 3501). Additionally, in order to test short-term cessation rates and explore linkages between cessation and pharmacotherapy, we limited this study to those over 18 years of age who were ready to quit in the next 30 days or who had begun an initiation attempt within 5 days before enrollment. Noneligible parties were directed to the publicly available version of smokefree.gov that did not collect any information from visitors.

**Figure 1:**
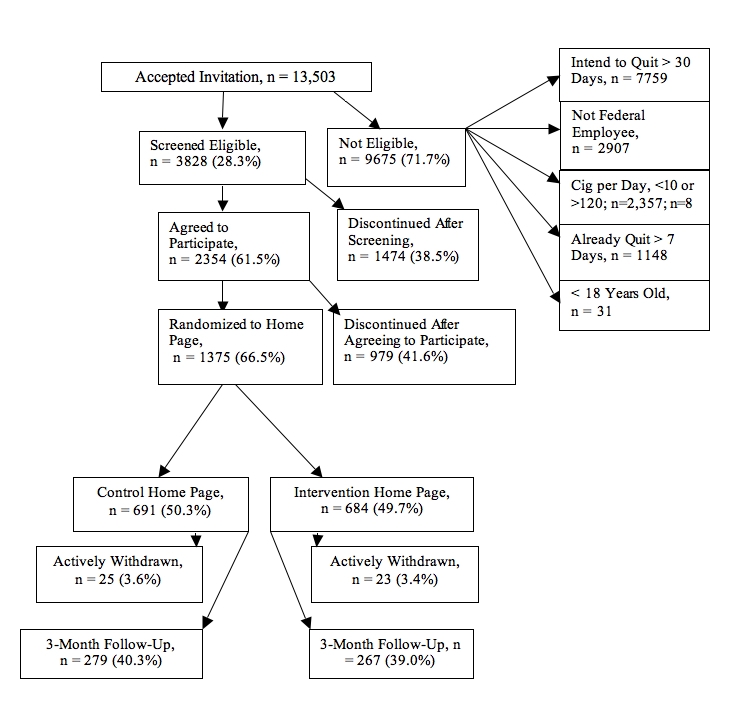
CONSORT flow diagram

Those eligible for participation were directed to a Consent/Study Description section that used an active consent format on three separate pages. Consenting participants were asked to provide a contact email, to choose an ID and password, and to complete a baseline questionnaire asking about demographics, history of nicotine/tobacco use, previous treatments for cessation, and a quit date. Once these steps were completed, participants were randomized to the publicly available version of smokefree.gov, designated as usual care (UC condition), or an identical-looking website that included an asynchronous bulletin board (BB condition). Randomization occurred via a computer algorithm (ie, random number generator) that selected from ID numbers generated with returned baseline questionnaires. Participants were told that they would be randomly assigned to 1 of 2 experimental conditions of the website and that the efficacy of both was unknown. The research team was also blinded to the assigned condition. Those in the BB condition were required to enter a username and password when posting messages. We have illustrated the flow-through of participants from beginning to end in Figure 1.

### Data Collection

Study enrollment, including informed consent and all data collection, occurred via secure (encrypted) Web transmission using SSL software. Data were transmitted in only one direction—from the participant to the study database, which could only be accessed by the study team. All identifying information was stripped from the summary datasets.

Follow-up questionnaires included items about smoking abstinence, satisfaction with the resources provided (“Did you find the resources on the website useful?” 0 = not at all, 4 = extremely useful), use of other cessation aids during the study period (see Table 2), and extent of perceived social support. Participants were asked, “Since you signed up for the Smokefree study, was there someone you had frequent contact with who has been supportive of your efforts to quit smoking?” Participants who answered “yes” were then asked, “How supportive was that person?” Responses were given using a Likert scale, where 0 = not at all supportive and 4 = very supportive. Finally, participants were asked, “Since using the website, are you now more motivated to quit smoking?” Again, we used a 5-point Likert scale, where 0 = not at all and 4 = very motivated.

### Interventions

**Figure 2:**
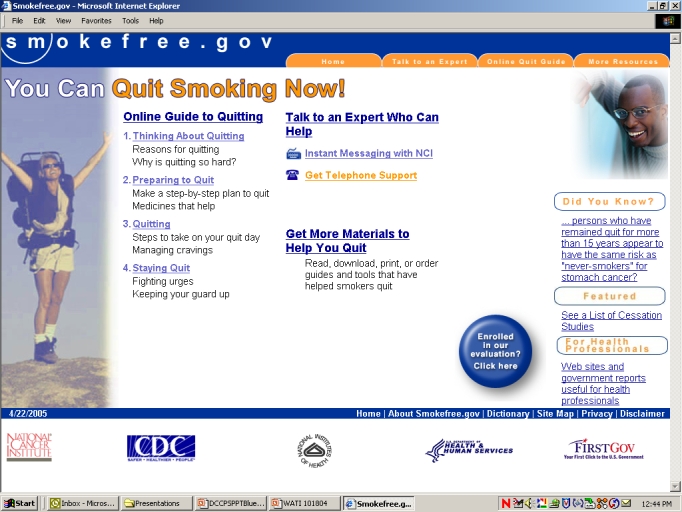
Screenshot of the smokefree.gov home page, UC condition

**Figure 3:**
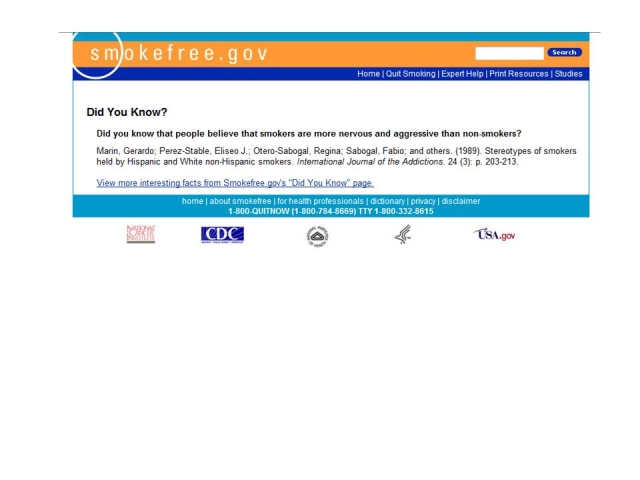
Screenshot (a) of the “Did You Know?” messages

**Figure 4:**
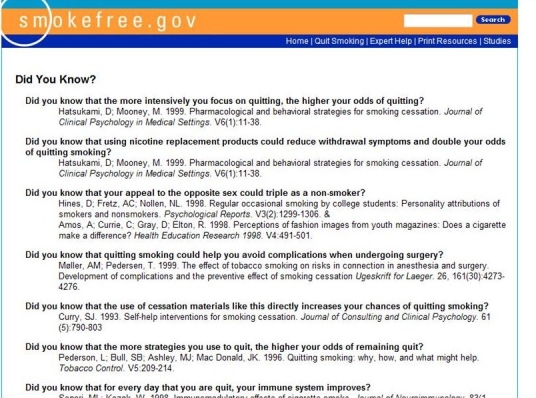
Screenshot (b) of the “Did You Know?” messages

The basic content in both conditions is shown in Figure 2 and is as follows: (1) online quit guide and 5 unique self-help materials targeted to specific populations, all shown to be effective in previous studies with smokers [17-21], (2) links for reaching a cessation counselor for one-on-one help either by telephone or instant messaging, (3) an interactive list of clinical trials still recruiting smokers who wish to quit smoking, (4) an interactive smokers risk tool showing changes in the risk of death due to smoking based on the smoker’s history and time of quitting, and (5) a series of empirically based statements about positive health changes that commonly follow cessation (see Figure 3 and Figure 4). The BB condition offered a forum where participants could respond to some seeded categories posted on the board or start their own message.

All participants, regardless of condition assignment, received email reminders (eg, Quit Date Reminder, Follow-up Survey Reminder). During the first 2 weeks of the study, a period identified with the highest rates of relapse and study dropout, participants received 4 email reminders, unless they indicated in a previous email that they wished to discontinue their participation. Each email contained tips on quitting, a brief message encouraging use of the website, as well as the time frame of the future follow-up assessments. For example, a few days after enrollment, participants received an email that included their score on the nicotine dependence quiz along with a link to text on the website related to nicotine dependence. A subsequent email included information about health improvements (eg, lung function) associated with quitting after a certain amount of time and links to content that discussed commonly used medications.

### Time Spent on Website

We calculated utilization by summing the time each participant spent on each page of the website. This calculation under-represents actual usage because at least a third of the resources listed for both groups contained links that took users outside the website (eg, live help, telephone help by state, clinical trials, certain self-help guides). Therefore, the data presented on site utilization may be a substantial under-reporting of actual total time participants spent on the website.

### Three-Month Follow-Up

Three months after the quit date, participants were sent up to 2 emails with a link to the follow-up questionnaire. Those not completing the follow-up after 2 reminders were mailed the questionnaire with a postage-paid return envelope and a thank you note containing either a prepaid calling card worth 100 minutes within the continental United States or US$7.40 worth of postage stamps for the added inconvenience of completing mailed forms. Those not returning the mailed questionnaire were called up to 5 times for their responses.

### Data Analysis

We used chi-square tests to test for associations between categorical variables (eg, yes/no) and 2-sided *t*tests to compare differences between relevant independent variables (eg, minutes of utilization). We used odds ratios along with 95% confidence intervals to express the proportion of nonsmokers for variables of interest (eg, days until quit date, medication use). Since there were no differences in participant characteristics across phases or experimental conditions, we aggregated the phase I and phase II data and collapsed across groups for the reported outcomes. We report both the ITT analysis, which treats all baseline participants who do not complete a follow-up survey as smokers, and the adherence analysis, which includes those who took part in the follow-up survey.

## Results

### Participant Demographics

Results from the analysis of the baseline demographics and smoking behavior are presented in Table 1. Initial analyses revealed no differences between treatment groups, so data are presented in aggregate form. Just under half of our sample were men (46.1%, n = 634). The average age of participants was 43.6 years. About half of the group had attained some college education (49.2%), with 12.9% attaining only high school (or the equivalent) or less. Most participants were non-Hispanic White (69.1%), with 16.9% non-Hispanic Black and 7.0% Hispanic.

 The average cigarette use was just under a pack a day (18.3 cigarettes per day), and the average smoker reported a “moderate” dependence on cigarettes, with a score of 4.5 on the Faggerstrom Test of Nicotine Dependence (FTND). Most of the sample (68.4%, 929/1371) smoked within 30 minutes of waking, and 26.3% (n = 367) did so within the first 5 minutes after waking. Nearly all participants (94%) reported having made a previous quit attempt. On average, smokers rated their confidence in their ability to quit as 3.2/5.0, or moderately confident.

Baseline demographic characteristic were examined in relation to use of our website to determine whether or not it was appealing to as broad a cross-section of federal employees as possible, including racial minorities and those from diverse socioeconomic backgrounds. Demographics did not differ by experimental condition. Only 1 baseline characteristic predicted increased use of the website: lower annual income (*P* = .001).

**Table 1:** Participant demographics (n = 1375)

Variable	%^a^	No.
Female gender	53.9	741
Age, mean (SD)	43.6 (10.3)	1375
**Education, highest level completed**		
High school/GED^b^ or less	12.9	176
Some college/Associate of Arts degree	49.2	671
College graduate	24.0	328
Post-graduate degree	13.9	190
**Annual household income (US$)**		
< 34,999	12.4	167
35,000 to 49,999	21.0	283
50,000 to 74,999	26.8	362
75,000 to 99,999	20.5	277
≥ 100,000	19.3	261
**Ethnicity**		
Hispanic	7.0	95
Non-Hispanic White	69.1	934
Non-Hispanic Black	16.9	228
**Tobacco Dependency**		
Cigarettes per day baseline, mean (SD)	18.3 (8.5)	1375
Nicotine Dependence (FTND), mean (SD)	4.5 (2.3)	1366
Age of first cigarette, mean yrs (SD)	16.1 (4.4)	1372
Age became a regular smoker, mean yrs (SD)	19.3 (5.3)	1369

^a^Numbers are percentages unless otherwise indicated.

^b^General Educational Development (equivalent of high school diploma).

### Past and Concurrent Use of Cessation Aids

As with baseline variables, no differences were found between conditions regarding cessation aids, so data are presented for the combined groups. As shown in Table 2, use of pharmacotherapy during past cessation attempts and the current attempt was high. The majority of the participants had tried nicotine replacement therapy (NRT) in the past, and about half were using NRT during the current study. This was followed by some form of assistance through the Internet (35.7%), with 10.3% of that help from another cessation website. The least used types of help were the nicotine nasal spray and quitlines.

**Table 2:** Cessation aids used in the past and during the study period

Type of Cessation Aid	Use in Past (n = 1291)		Use During Study (n = 522)^a^	
	%	No.	%	No.
**All Medication**	77.5	1000	51.9	271
All NRT	71.3	921	43.3	226
Patch	58.7	758	28.0	148
Nicotine gum	41.7	538	15.7	82
Nicotine lozenge	10.0	129	0.8	42
Nicotine inhaler	6.4	83	2.1	11
Nicotine nasal spray	0.9	11	0.2	1
Zyban	34.9	450	15.3	80
Other antidepressants	6.7	86	3.3	17
**Internet**	35.7	460	NA	NA
Other cessation websites	10.3	133	5.7	30
Chat room/BB	6.8	88	NA	NA
**Other**				
Self-help materials	19.7	254	9.0	47
Hypnosis/acupuncture	17.0	219	2.1	11
Group/individual counseling	13.3	172	2.9	15
Other cessation materials	12.9	167	6.3	33
Quitlines	1.4	18	1.0	5
No cessation help	15.0	194	34.0	179

^a^NA = not asked.

### Utilization of Pages

We were limited in our comparisons of popular requested website pages because some of the features consisted of other National Institutes of Health and US Health and Human Services resources that required external links, such as telephone and text messaging support and studies looking for participants. However, within the pages that were internally hosted (between 150 and 196 pages depending on condition), 8 of the top 10 most visited pages on our website were from an HTML version of the National Cancer Institute’s guide “Clearing the Air,” which we labeled the “Online Guide to Quitting.” Visits to these pages did not notably differ between the UC and BB conditions, except for very minor differences in the ranking positions (see Table 3). The leading topics viewed from the guide included Preparing to Quit, Initial Phases of Quitting, and Nicotine Addiction.

**Table 3:** Usage of tools (pages) on smokefree.gov, by condition

BB Condition	Hits	UC Condition	Hits
Guide/preparing_to_quit.html	437	Guide/nicotine_addiction.asp	413
Guide/initial_phases.html	389	Guide/preparing_to_quit.html	400
Guide/nicotine_addiction.asp	388	Guide/initial_phases.html	360
Guide/staying_quit.html	314	Index.asp	320
Guide/medicines.html	307	Guide/medicines.html	298
Guide/considering_quitting.html	299	Guide/considering_quitting.html	287
Index.asp	295	Guide/staying_quit.html	265
Info.html	281	Info.html	264
Guide/withdrawal symptoms.html	271	Guide/withdrawal symptoms.html	251
Pop_triggers.asp	242	Pop_triggers.asp	240

### Time Spent on the Website

Table 4 presents data for time spent on the website by various subgroups of users. For the pages on the website that did not take people outside of smokefree.gov, the average number of minutes spent for either condition was 14.4 minutes (n = 1083). This calculation excluded time devoted to answering any questionnaire items. Time on the website was higher for those assigned to the BB condition versus the UC condition and was nearly double for those who returned a follow-up questionnaire (vs dropouts), those who reported abstinence (vs still smoking), and those who made a serious quit attempt by abstaining for at least 24 hours (vs those who did not). The longest time spent on the website (30 minutes) was for those whose quit attempt began in the 5 days prior to registering for the study. Past or present use of medication did not influence time spent on the website.

**Table 4:** Time (in minutes) spent on website, by study participation and quitting behaviora

	Yes (min, No.)	No (min, No.)	*t* test
Assigned to BB condition	18.0, 526	11.1, 557	*t*_1081_ = 2.5, *P* = .01
Returned follow-up questionnaire	21.2, 456	9.6, 627	*t*_1081_ = 3.7, *P* < .001
Serious quit attempt (smokers)	22.4, 260	10.4, 730	*t*_988_ = 2.3, *P* = .02
7-day abstinence at 3 months	23.4, 82	13.8, 1000	*t*_1081_ = 2.6, *P* = .01
Quit attempt before enrollment date	29.4, 77	12.5, 304	*t*_379_ = 3.6, *P* = .001

^a^Sample size varies based on complete records for both minutes of use and the variable reported.

### Bulletin Board Use and Smoking Cessation

Among those assigned to the BB condition, only 242 opted to look at the Bulletin Board feature by clicking on the link, and of those visiting the link, only one third (81/242) either selected an individual message to view or posted a message. This low utilization rate (81/684, or 11.8%) limited our ability to analyze the impact of bulletin board use on cessation.

### Smoking Cessation and Reduction

In Table 5, we present cessation outcomes across experimental conditions 3 months after enrolling in the study. When counting nonresponders (63%, n = 829) as smokers (ITT), 6.8% of participants said that they had been quit for 7 consecutive days. When making no assumptions about nonresponders (adherence sample), 17% said that they had quit smoking. When limiting our analysis just to smokers who had initiated a quit attempt during the 5 days before study entry or on the day of entering the study, the ITT quit rate 3 months later was 29.6%. With our adherence sample, this quit rate was 44.4%. Outcomes did not significantly differ by condition. For participants who were still smoking and who also completed follow-up (n = 339), the number of cigarettes smoked per day dropped from 17.8 to 13.1, which was statistically significant (*t*_338_= 12.3, *P* < .001). This did not differ by condition. The change in cigarettes per day was significant for both groups (UC: 17.6 to 12.8, n = 177, *t*_176_= 9.3, *P* < .001; BB: 18.0 to 13.5, n = 162, *t*_161_= 8.0, *P* < .001).

**Table 5:** Abstinence rates among respondents, by ITT or adherence sample

Variable	Abstinent (7-Day)^a^			
	All	UC	BB	*P*
	% (n/N)	% (n/N)	% (n/N)	
**ITT**	6.8 (93/1375)	6.9 (48/691)	6.6 (45/684)	.79
Quit within 5 days before study	29.6 (24/81)	35.1 (13/37)	25.0 (11/44)	.33
**Adherence sample**	17.0 (93/546)	17.2 (48/279)	16.9 (45/267)	.91
All medication/users	19.9 (54/271)	18.7 (25/134)	21.2 (29/137)	.61
NRT users	21.2 (48/226)	19.7 (23/117)	22.9 (25/109)	.55
Quit within 5 days before study	44.4 (24/54)	48.1 (13/27)	40.7 (11/27)	.59

^a^n = number abstinent, N = number within subgroup.

### Social Support

Nearly 80% of participants reported having a lot of social support for their quitting effort (79.4%, 965/1216). Extent of support felt by participants did not differ by experimental condition (UC: 78.0%, 475/611; BB: 81.0%, 490/605, *P*= .14); 14.5% (176/1216) of participants said they felt somewhat supported in their quit efforts, and only 3.5% (42/1216) said that they had little or no support, with 2.7% (33/1216) undecided.

### Satisfaction and Motivation to Quit

For those providing a follow-up questionnaire, the vast majority said that the website was useful (87.6%, 446/509) and that they were more motivated to quit smoking after having used the intervention (81%, 419/517). Those who reported being satisfied with the website did not differ by experimental condition (UC: 90.2%, 238/264; BB: 84.9%, 208/245, *P*= .08) nor did those who said that they were more motivated to quit (UC: 82.0%, 219/267; BB: 80.0%, 200/250, *P*= .58). This was contrary to our expectation as we expected that those in the BB condition would rate the website more favorably than those in the UC condition.

## Discussion

This RCT-based pilot study assessed utilization of, satisfaction with, and impact of 2 versions of smokefree.gov among smokers who wanted help with quitting and who worked for the federal government. The average length of time spent on the website was underestimated because only about three quarters of the content was internally hosted. Despite this, the average time on the website and the satisfaction with materials (among followed participants) was high compared with similar public health websites [22], particularly for those who stayed in the study or made a serious quit attempt. This was true regardless of experimental condition. Time on the website was longest for those who stayed in the study, for those who quit at 3 months, and for those who made a serious quit attempt either in the 5 days prior to the study or afterward. This suggests that interest in our materials was heightened for those in the early stages of cessation, when self-help materials may be particularly relevant [23]. Given the strong effect that having made a recent quit attempt had on outcomes in this study, future Web-based research should consider continuing to include this group. This will help to facilitate comparisons across studies while benefiting a group of smokers who appeared to be actively searching for cessation support.

The observation that nearly 90% of followed users reported satisfaction with the website and that 80% reported greater motivation to quit smoking after using the site is consistent with other research highlighting the website’s quality [24]. However, we are mindful that satisfaction with the website and motivation are lower among those who opted not to use it or to discontinue participation in the study.

While our sample had higher income and education than smokers generally do, it was more racially diverse than those from other large US studies and had greater gender parity [23,25] as well as higher use among participants from lower socioeconomic levels. This could be related to the more varied materials on the website, including the self-help guide written in Spanish (“Guia para Dejar de Fumar”) and the one developed for African American smokers (“Freedom from Smoking”). Additionally, as our sample was comprised of federal employees, and many government employment positions strongly encourage applicants from diverse backgrounds, we may have reached a more diverse group of smokers than many other studies simply due to the recruitment approach used in the current study [7,11,12].

Across all cessation materials on the site, the most frequently used was the HTML “Online Guide to Quitting,” which included topics known to be of greatest interest in the earliest days of the quitting process. Some of the selections (eg, medications, withdrawal symptoms) informed our decision to later expand these content areas in subsequent updates to the site.

Abstinence rates and smoking reduction reported in this study are comparable to other online interventions [7,8,11,26]. One study had better outcomes, with an 18% ITT quit rate [27], but recruited its participants through existing online smoking cessation support groups and among smokers who sought help for cessation online. This study also provided incentives for follow-up. This recruitment method contrasts sharply with ours, which used a largely disseminated email to federal employees within different government departments to explore and evaluate content, and which did not promise any incentive.

The high use of medication during the study had a small (4%) but favorable effect on cessation. Outcomes for those using medication in our study were similar to those reported by Strecher et al [11], who reported outcomes only for NRT users. Given the high rate of medication use among smokers willing to quit, future studies should consider reporting outcomes for those using/not using medications rather than attempt to exclude these users.

Despite research showing a strong interest in a bulletin board or similar feature [7], including our internal surveys with federal employees [16], actual use of this tool was low. A number of possibilities could account for this. First, users knew that they were taking part in a formal study that monitors site activity and were reminded of this each time they had to re-enter log-in information to view or post messages. This may have had inhibited use. Second, responses to postings generally occurred with a few days of the initial post rather than a few hours, as has taken place in other settings [22]. The delayed response to messages may have limited meaningful exchanges and discouraged individuals from posting additional messages*.* Third, it may be that individuals who anonymously search out help for their cessation are disinclined to use a social support tool [13] or already have a support network, such as the vast majority of participants in our study. Fourth, other research has shown that former smokers are significantly more likely to become active members of a bulletin board community than those who are planning a quit attempt [12]. Perhaps those who have already achieved some level of abstinence are less fearful of failure and more willing to share messages of encouragement compared to those who are at the beginning of the quitting process. Finally, bulletin board communities may require a minimum threshold of activity that is much larger than is possible with the number of participants we recruited. In general, a much better understanding of the conditions that lead to demand for this feature, particularly in light of the popularity of this feature in other settings, is needed.

In considering appropriate methodology for assessing Web-based features and the efficacy of Web-based interventions within real-world contexts, there are a number of challenges. Some researchers have argued that it is important to exclude smokers who are participating in any other forms of cessation help in order to detect the independent effect of a website intervention on cessation. However, attempting this would be both impractical and potentially unethical if those other forms of cessation assistance are effective. In the real world, in which the role of Internet searches and use is increasing, successful cessation often means employing a number of strategies simultaneously. Of significant concern is that attrition rates are extremely high, both in our study and in those throughout the field of WATI research, where anonymity is emphasized. It is possible that we could have obtained a higher response rate with additional emails or calls. However, we were mindful that attrition can actually increase when participants are contacted more frequently than ours were [14].

### Limitations

This study has several limitations. First, the population was made up of government employees and contractors with above-average education and therefore is not necessarily generalizable to the general population of smokers online. We were limited to surveying only government employees or contractors due to restrictions that limit burdening the public with surveys. Second, similar to many other eHealth interventions [13], our attrition was high despite use of multiple methods of follow-up. Future studies may wish to highlight the importance of collecting follow-up data with participants before they sign up for the intervention. Third, we cannot rule out possible contamination between conditions. To limit the number of occurrences where this may have taken place, we examined the records of all baseline questionnaires entered on any given day and looked for similar demographics, smoking histories, usernames, and time of entry. We found only one instance where entries were suspicious (same date, similar time, and similar demographics/smoking history) and then removed this record from the dataset. Fourth, we used a very primitive measure of website use/utilization, which did not include more sophisticated methods that have been used in the field [7,28].

### Conclusions

Despite these limitations, our results point to the importance of an individual’s engagement in the treatment process and available resources. We found that smokers who fully participated in the evaluation of smokefree.gov and used the site were satisfied with the content provided. Use (measured by time) was highest for those who quit on, or a few days prior to, the study start date. Given that most WATI studies simply ask smokers to answer “yes/no” to the question of whether or not they are ready to quit smoking, it is very likely that those included for study have already begun such an attempt. Future studies should ask about any recent quit attempts (eg, within a few days) as well as the willingness of the participant to utilize treatment materials. Both factors seem critical to treatment engagement and future success in abstinence. As noted previously, the potential impact of Web-based interventions for which effects will be small lies in the extensive reach of the Internet and its ability to reduce obstacles to treatment availability.

## Abbreviations

ACS: American Cancer Society
BB: bulletin board condition
FTND: Faggerstrom Test of Nicotine Dependence
NRT: nicotine replacement therapy
RCT: randomized controlled trial
UC: usual care condition
WATI: Web-assisted tobacco intervention
